# Prevalence of unmet need for family planning and unintended pregnancies among women of reproductive age living with HIV in sub-Saharan Africa: a systematic review and meta-analysis

**DOI:** 10.4314/ahs.v24i2.6

**Published:** 2024-06

**Authors:** Hafidha M Bakari, Oluwafemi Alo, Mariam S Mbwana, Swalehe M Salim, Emilie Ludeman, Taylor Lascko, Habib O Ramadhani

**Affiliations:** 1 President's Office Regional Administration and Local Government, Dodoma, Tanzania; 2 Maryland Global Initiative Cooperation, Abuja, Nigeria; 3 Primary Health Care Institute, Iringa, Tanzania; 4 Canada World Youth; 5 Health Sciences and Human Services Library, University of Maryland Baltimore, Baltimore, USA; 6 Center for International Health, Education, and Biosecurity, University of Maryland School of Medicine, Baltimore, MD, United States; 7 Institute of Human Virology, University of Maryland School of Medicine, Baltimore, MD, United States

**Keywords:** Unmet need for family planning, women living with HIV, un intended pregnancy, sub-Saharan Africa

## Abstract

**Introduction:**

Family planning is an effective intervention for women living with HIV who do not desire to have children to reduce vertical transmission and infant- and pregnancy-related mortality.

**Objectives:**

We aimed to evaluate the prevalence of unmet need for family planning (UFP) and unintended pregnancies among women living with HIV in sub-Saharan Africa.

**Methods:**

This was a systematic review that searched databases from March 2007 to December 2021. UFP was defined as women who were sexually active and did not desire to have additional children (unmet need for limiting), or who delayed their next pregnancy (unmet need for spacing) but were not using any contraception. Unintended pregnancies were defined as women who reported that their last pregnancy was unintended. Forest plots were used to present the pooled prevalence with a 95% confidence interval (CI).

**Results:**

Total of 35 articles were included. Overall, the pooled prevalence of UFP was 30.1% (95%CI, 26.4–33.9). The pooled prevalence of unmet need for spacing was 11.9% and 14.2% for limiting.. The pooled prevalence of unintended pregnancy was 16.5% (95%CI, 9.4–25.1).

**Conclusion:**

Three in ten women of reproductive age living with HIV in Africa have UFP. Efforts to prevent unsafe abortions from unintended pregnancies are needed to minimize the UFP.

## Introduction

By the end of 2021, there were an estimated 36.7 million adults (15-49 years) living with HIV globally, with women accounting for 19.7 million^1^. Vertical transmission of HIV is the predominant route of HIV acquisition among children. It is estimated that about 1.3 million women living with HIV become pregnant each year and without effective intervention to reduce vertical transmission, 15-45% of HIV infections will occur in newborns^2^. Although antiretroviral therapy (ART) has profound benefits of reducing infant HIV infection to less than 2% among women living with HIV, effective family planning is equally important in women living with HIV who do not desire to have children^3^.

Prior studies have estimated that, if all women in sub-Saharan Africa who did not wish to become pregnant could have access to contraceptive services, 333,000 new infant infections could be averted annually (4, 5). Data from 15 President's Emergency Plan for AIDS Relief (PEP-FAR)-supported countries showed that contraception averts nearly 200,000 new infections (6). Moreover, an estimated annual cost saving in preventing unintended HIV-positive births ranged from $26,000 to $2.2 million (6). The World Health Organization estimated that 61% of all unintended pregnancies end up in induced abortions with 45% of these abortions being unsafe (7). Furthermore, developing countries account for 97% of all unsafe abortions and up to 13% of maternal mortality is attributed to unsafe abortions (7, 8). These data underscore the need to advocate for uptake of family planning methods for those in need.

Despite individual and public benefits of family planning, as well as the prevention of HIV transmission to infants, over 200 million women have an unmet need for family planning in low- and middle-income countries (9). Several factors have been documented to be associated with unmet need for family planning among women living with HIV, including low education level, husband's disapproval of family planning, number of living children, high household income, fear of contraceptive side effects, desire to have children, and not having knowledge of vertical transmission(10-12). Discussions with health care providers, couples jointly making healthcare decisions, awareness of family planning methods, and having a partner who is not infected with HIV are associated with decreased risk of unmet need for family planning (10-16).

Given its potential benefits, such as reduction of vertical transmission, pregnancy-related maternal mortality, appropriate children spacing, aversion of unintended pregnancies, reduction in total and unsafe abortions, and reduction in infant and childhood mortality (17, 18), it is critical to understand the magnitude of unmet need for family planning among women living with HIV. Data from different studies have indicated regional variation in the prevalence of unmet need for family planning. This study aims to estimate pooled prevalence of both unmet need for family planning and unintended pregnancies among women living with HIV in sub-Saharan Africa. Understanding the magnitude of these outcomes may increase focus on interventions to minimize low uptake and maximize potential benefits of family planning among women living with HIV in these settings.

## Methods

### Registration

This systematic review has been registered in the International Prospective Registry of Systematic Review with registration number CRD42023393600. This systematic review and meta-analysis adhered to the Preferred Reporting Items for Systematic Reviews and Meta-Analysis (PRISMA) statement.

### Search strategy

PubMed, Cochrane CETRAL, Embase and Google Scholar databases were searched between March 2007 to December 2021. Search terms were used to capture concepts of unmet need for family planning and prevalence of unintended pregnancy among women of reproductive age living with HIV in sub-Saharan Africa. The search was restricted to papers written in English. Two independent authors searched for the manuscripts. Full search strategies are available in the appendix 1.

### Inclusion criteria

Observational studies that involved women of reproductive age (15-49 years) living with HIV in sub-Saharan Africa, reported unmet need for family planning, and written in English were eligible for inclusion. We excluded studies that did not report unmet need for family planning.

### Study selection, quality assessment and data abstraction

Databases were searched and results were placed in Covidence software. Covidence was also used to remove duplicate references. The National Institutes for Health (NIH) tool was used to assess the quality of studies (19). Two review authors (HBM and HRO) completed the study selection for inclusion in the appraisal process. Disagreement between two independent reviewers for the inclusion of the manuscripts was handled by the third reviewer (MM). Using an Excel spread sheet, two review authors (HBM and HRO) abstracted the following data elements from the included studies: authors, year of publication, country in which the study was conducted, study design, study period, outcomes, and sample size.

### Outcome

The main outcome of interest was the prevalence of unmet need for family planning as defined by the World Health Organization (20). Unmet need for family planning is categorized in two forms, unmet need for limiting and unmet need for spacing children. Unmet need for family planning was defined as women of reproductive age who were sexually active and did not desire to have additional children (unmet need for limiting), or who desired to delay their next pregnancy (unmet need for spacing) but were not using any contraceptive methods. We also examined prevalence of unintended pregnancy computed as number of women who reported their last pregnancy was unintended per total number of women evaluated in the study

### Analysis

We used forest plots to explore prevalence of unmet need for family planning among women of reproductive age living with HIV in sub-Saharan Africa. A random effects model with an I2 statistic was used to account and test for study heterogeneity. Finally, pooled prevalence of unmet need for family planning and prevalence of unintended pregnancy was computed. In addition, prevalence of unmet need for family planning was also presented by geographic regions and year at which the studies were conducted. Chi-squared tests were used to assess differences in prevalence of unmet need for family planning in these stratified analyses. The publication bias was assessed using the Egger regression asymmetry test. For both heterogeneity and publication test, a p-value < 0.05 indicated the presence of heterogeneity and publication bias respectively. All statistical tests were performed using STATA version 16 (Stata Corporation, College Station, Texas, USA).

## Results

**Figure 1:**
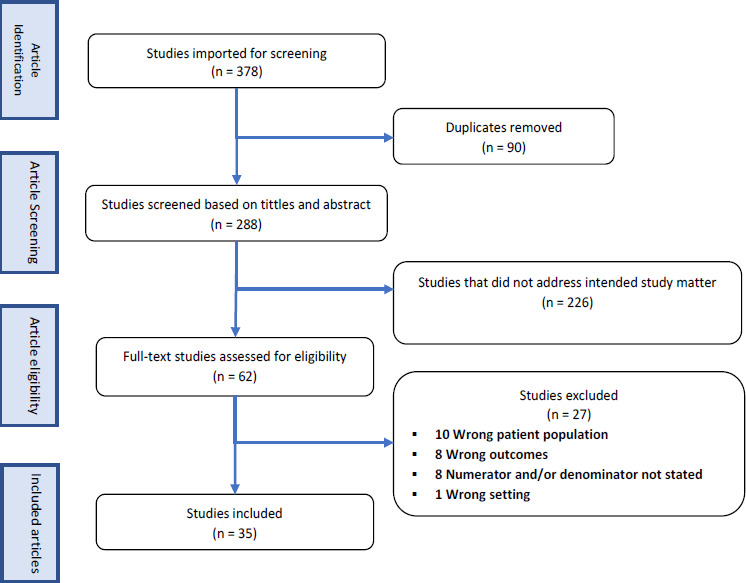
PRISMA flow diagram of the included studies for meta-analysis of unmet need for family planning among women of reproductive age living with HIV in sub-Saharan Africa

A total of 378 publications were retrieved through searches and 90 were duplicates and removed, leaving 288 publications to have their titles and abstracts screened and 226 were excluded as they did not address the intended study matter. The remaining 62 received a full review and 35 were eligible for final analysis (Figure 1). Of the 35 studies, 33 (94.3%) reported prevalence of unmet need for family planning while 2 reported unmet need for child limiting only.

### Study selection

#### Characteristics of studies included

This analysis included both published and unpublished articles. Study designs for articles included cohort (n = 3) and cross sectional (n = 32). Sample size for studies ranged from 94 to 1,631. A total of 18,500 women of reproductive age living with HIV were included in this analysis (Table 1). We included studies from eastern Africa (n = 21), western Africa (n = 9), southern Africa (n = 4), and central Africa (n = 1).

**Table 1:** Summary of characteristics of the included studies

Author and publication year	Study period	Country	Study design	Sample size n	Unmet need for Family Planning %	Unmet need for Spacing %	Unmet need for Limiting %	Quality scores %
Abeje et al, 2016(14)	2013	Ethiopia	Cross-sectional	530	24.6	-	-	100.0
Abubekar et al, 2019(16)	2016	Ethiopia	Cross-sectional	334	25.1	16.2	9.0	90.0
Arikawa et al, 2020(21)	2015	Côte d'Ivoire	Cross-sectional	1631	39.7	-	-	80.0
Dejene et al, 2021(22)	2020	Ethiopia	Cross-sectional	409	33.0	18.5	14.7	90.0
Demissie et al, 2021(23)	2018	Ethiopia	Cross-sectional	654	15.9	-	-	90.0
Edward et al, 2021(24)	2021	Kenya	Cross-sectional	347	21.0	3.0	18.0	90.0
Olanrewaje et al, 2021(25)	2015	Nigeria	Cross-sectional	933	-	-	21.9	80.0
Ezugwu et al, 2014(26)	2012	Nigeria	Cross-sectional	400	26.8	-	-	90.0
Feyisa et al, 2014(27)	2014	Ethiopia	Cross-sectional	401	15.5	7.5	8.0	80.0
Feyisa et al, 2020(28)	2018	Ethiopia	Cross-sectional	360	25.0	-	-	80.0
Habte et al, 2015(29)	2010	Malawi	Cross-sectional	489	21.9	7.4	14.5	80.0
Kasete et al, 2018(30)	2014	Ethiopia	Cross-sectional	451	32.4	18.0	14.4	90.0
Kassie et al, 2021(31)	2018	Ethiopia	Cross-sectional	441	24.5	15.4	9.1	90.0
Laryea et al, 2014(32)	2012-2013	Ghana	Cross-sectional	230	27.8	-	-	90.0
Makumbi et al, 2010(33)	2008-2009	Uganda	Cross-sectional	998	34.8	-	-	80.0
Yotebieng et al, 2015(34)	2011-2012	DRC	Cohort	699	17.6	-	17.6	85.0
McCoy et al, 2014(35)	2012	Zimbabwe	Cross-sectional	584	18.5	-	-	90.0
Mekdes et al, 2015(36)	2015	Ethiopia	Cross-sectional	658	19.1	13.2	5.9	90.0
Mohammed et al, 2020(37)	2000	Nigeria	Cross-sectional	325	35.1	18.8	16.3	80.0
Mutiso et al, 2008(38)	2008	Kenya	Cross-sectional	94	55.8	-	-	80.0
Ngugi et al, 2014(39)	2012	Kenya	Cross-sectional	137	31.0	-	-	80.0
Okigbo et al, 2014(40)	2008-2009	Nigeria	Cross-sectional	529	38.9	-	-	80.0
Okunola et al, 2019(41)	2015	Nigeria	Cross-sectional	425	20.0	2.6	17.4	80.0
Oyebode et al, 2016(42)	2016	Nigeria	Cross-sectional	350	51.6	-	-	83.0
Rucinski et al, 2018(43)	2009-2011	South Africa	Cohort	850	58.8	-	-	85.0
Schaan et al, 2014(44)	2009	Botswana	Cross-sectional	155	37.6	-	-	80.0
Schwartz et al, 2012(45)	2009-2010	South Africa	Cohort	850	28.0	-	-	92.0
Thindwa et al, 2019(46)	2015-2016	Malawi	Cross-sectional	578	35.0	-	-	80.0
Tim et al, 2007(48)	2004	Lesotho	Cross-sectional	756	31.3	-	-	80.0
Tusubira et al, 2020(49)	2016	Uganda	Cross-sectional	369	33.0	24.3	8.6	90.0
Wanyenze et al, 2015(50)	2015	Uganda	Cross-sectional	512	38.0	17.6	20.4	90.0
Wekesa et al, 2015(51)	2009-2010	Kenya	Cross-sectional	318	39.6	6.1	33.5	90.0
Yaya et al, 2020(52)	2016	Togo	Cross-sectional	461	-	-	9.0	90.0
Zewdie et al, 2020(15)	2018	Ethiopia	Cross-sectional	518	35.3	-	-	90.0

#### Prevalence of unmet need for family planning

**Figure 2:**
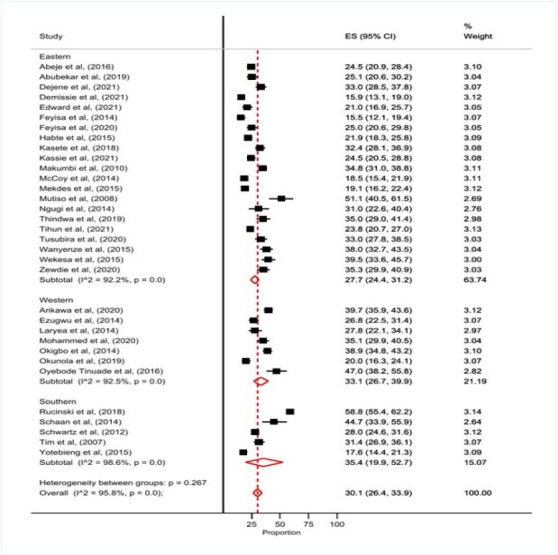
Prevalence of unmet need for family planning among women of reproductive age living with HIV in Africa 2007-2021

The overall, pooled prevalence of unmet need for family planning was 30.1% (95% CI 26.4-33.9) (Figure 2). There were both heterogeneity and publication bias as noted by a p-value of <0.001 for the I2 statistic and Egger test, respectively. Fourteen and 17 manuscripts reported unmet need for spacing and limiting, respectively. Pooled prevalence of unmet need for spacing was 11.9 (95% CI 8.6-15.6) and 14.2 (95% CI 11.4-17.3) for limiting.

#### Stratified analysis

There were regional variations on the prevalence of unmet need for family planning with the lowest prevalence reported in eastern Africa (27.7%) and highest prevalence in other regions including southern and central Africa, (35.4%) (Table 2). Compared to studies done before 2010, those done in 2010-2014, 2015-2019, and on/after 2020 had statistically significantly lower prevalence of unmet need for family planning, (37.1% vs 26.9%, p <0.001; 37.1% vs 29.1%, p <0.001; and 37.1% vs 27.3%, p < 0.001, respectively).

**Table 2:** Unmet need for family planning and unintended pregnancy among women of reproductive age living with HIV in sub-Saharan Africa

Variables	Number of studies	Total sample	Prevalence (95% C.I)	I^2^	P-value
Unmet need for family planning					
Overall Region	33	13,812	30.1 (26.4 - 33.9)	95.8	0.00
Eastern	21	8,607	27.7 (24.4 - 31.2)	92.2	0.00
Western	7	2,691	33.1 (26.7 - 39.9)	92.5	0.00
Others*	5	2,514	35.4 (19.9 - 52.7)	98.6	0.00
Year of study					
<2010	7	2,640	37.1 (32.2 - 42.0)	83.7	0.00
2010-2014	10	4,547	26.9 (18.2 - 36.7)	98.0	0.00
2015-2019	14	5,869	29.1 (24.5 - 34.0)	93.8	0.00
2020-date	2	756	27.3 (24.2 - 30.5)	-	-
Spacing	14	5,908	11.9 (8.6 - 15.6)	94.3	0.00
Limiting	17	7,631	14.2 (11.4 - 17.3)	92.6	0.00
Unintended pregnancy	16	6,767	16.5 (9.4 - 25.1)	98.7	0.00

* Southern (4) and Central region (1)

#### Prevalence of unintended pregnancy

**Figure 3:**
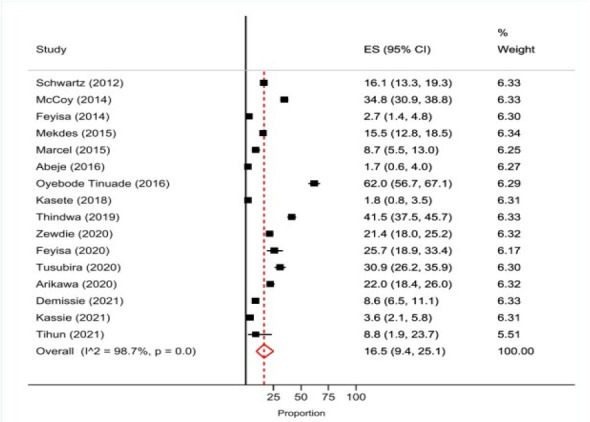
Prevalence of unintended pregnancy among women of reproductive age living with HIV in Africa, 2012-2021

Unintended pregnancy was reported by 16 studies. The pooled prevalence of women who reported their last pregnancy was unintended was 16.5% (95% CI 9.4-25.1). The lowest prevalence of unintended pregnancy was 1.7% (95% CI 0.6-4.0) and the highest reported was 62.0% (95% CI 56.7-67.1) (Figure 3).

## Discussion

We conducted a systematic review and meta-analysis among women of reproductive age living with HIV in sub-Saharan Africa to evaluate prevalence of unmet need for family planning and unintended pregnancy. The overall prevalence of unmet need for family planning and unintended pregnancy in these settings was approximately 30% and 17%, respectively. We observed pooled prevalence of unmet need for family planning declining over time with 37% for studies done before 2010 compared to 27% for those done on/after 2020. We also noted regional variations with 35% being the highest pooled prevalence of unmet need for family planning in southern and central Africa.

The overall unmet need for family planning in sub-Saharan Africa was higher than that ever reported in America (5.9%) and Europe (7.4%) (53). Although we observed a decline in unmet need for family planning over time, the UN Sustainable Development Goal to end unmet need for family planning is unlikely to be achieved by 2030 (53). In the effort to create a one stop shop where HIV and family planning services could be accessed at the same location, the World Health Organization recommended the integration of family planning services with HIV services (54). Following its implementation, current evidence shows a reduction in unmet need for family planning in healthcare facilities that adopted integration compared to healthcare facilities without integrated services (55). Although successful, several challenges of integration have been reported, including a low number of HIV healthcare providers with basic family planning training, the need for additional time given to providers in discussing family planning in addition to the provision of comprehensive HIV services, and long wait times for family planning services in addition to the already long waiting times for HIV services (56, 57). These challenges limit uptake and sustainability of family planning services.

While non-governmental organizations play an important role in improving access to family planning services, more efforts from country ministries of health are needed to minimize existing gaps in unmet need for family planning. Increasing the number of providers with basic family planning training and reduction of client waiting time may promote utilization of family planning services at facilities with integrated services. Proper referral systems to family planning services are critical to improve uptake of these services in facilities without integrated services. In addition, while most women living with HIV are made aware of family planning services after attending healthcare facilities, in this era of technology, social media platforms can be leveraged to disseminate family planning information, capturing more clients and improving uptake of family planning services. Prior systematic reviews have shown that women who were aware of family planning services were more likely to use them compared to those who were not aware (58).

Nearly two in ten women living with HIV had an unwanted pregnancy. Prior data has shown that prevalence of induced abortion among pregnant women living with HIV was 6.5%, compared to 2.9% among women living without HIV (59). For women living with HIV who knew their status prior to becoming pregnant, the prevalence of induced abortion was 20% compared to 14% among those were not aware of being infected prior to conception (60). These data indicate high burden of induced abortions among women living with HIV. Moreover, the World Health Organization reported that 61% of all unintended pregnancies end up in induced abortions with 45% of these abortions being unsafe (7). Data on induced abortions may also be under-reported due to the illegal nature of abortion services in many countries in sub-Saharan Africa(61, 62). Minimizing gaps in unmet need for family planning is critical to prevent unsafe abortions resulting from unintended pregnancies.

We are aware of other individual factors predominantly reported to be associated with low uptake of family planning services. These include husband disapprovals, low level of education, low perceived risk of pregnancy, and fear of experienced and perceived side effects (10, 58, 63). Qualitative studies conducted to understand barriers of uptake of family planning services revealed additional challenges, including family and social pressure to bear children, inconsistency across providers in family planning counselling, and lack of continuity in family planning counselling (64, 65). Furthermore, gender-based violence due to covert use of contraceptives, family conflicts over the use of modern contraceptives, and desire to shift to traditional methods are additional barriers to the use of family planning methods (66). A study in Zimbabwe also showed that providers were judgmental of clients about contraceptive use based on the client's age, misinformation about contraceptive use, and inadequate information about interactions between ART and contraceptives as barriers to family planning use (67). Overall, to minimize the gaps of uptake in family planning, addressing clients, healthcare providers, and healthcare system challenges is critical. For example, adequate male partner involvement and training on anticipated and perceived undesirable side effects may improve support and increase uptake of family planning services. It is critical to have non-judgmental healthcare providers and to introduce differentiated youth friendly family planning centers that offer services to youth who predominantly face judgement from healthcare providers. Enhancement of community involvement, strengthening of family planning counselling, and increasing investment in family planning is paramount to increase uptake. As efforts to overcome some of the challenging individual factors, improving facility-related factors and client education on the perceived side effects may be potential solutions to increase uptake of family planning services. Family planning is notable for reducing unwanted pregnancies, vertical transmission, unsafe abortions, poor maternal and child outcomes, short inter-pregnancy intervals, early childbearing, and physical abuse among women living with HIV (17, 18).

This systematic review predominantly involved cross-sectional studies, which limited our ability to provide estimates on the incidence of unwanted pregnancies. Studies on incidence are important as they contribute to our understanding of current trends in outcomes. The majority of the studies were conducted in eastern Africa, especially Ethiopia, which also limited extensive comparison to other regions. Furthermore, because the search was limited to publications written in English, it is likely that other relevant publications from non-English journals were missed. The main strength of this research is inclusion of many studies with pooled estimates from a large sample. This study remains relevant as it provides estimates of unmet need for family planning among women living with HIV in sub-Saharan Africa.

## Conclusion

Nearly three in ten women of reproductive age living with HIV in Africa have an unmet need for family planning and this proportion has been consistent for the past 15 years. To achieve the UN Sustainable Development Goal to end unmet need for family planning, minimizing barriers of uptake of family planning services is critical. Furthermore,, efforts to prevent unsafe abortions from unintended pregnancies are needed to minimize unmet need for family planning among women living with HIV.
